# Supplementary material for: Genetic continuity of Indo-Iranian speakers since the Iron Age in southern Central Asia
Source: Sci Rep. 2022 Jan 14;12:733. doi: 10.1038/s41598-021-04144-4 (PMC8760286; doi:10.1038/s41598-021-04144-4)
Supplement: Supplementary file 1 — Supplementary Information 1. [file 41598_2021_4144_MOESM1_ESM.pdf]

SUPPLEMENTARY MATERIAL

GENETIC CONTINUITY OF INDO-IRANIAN SPEAKERS SINCE THE IRON AGE IN SOUTHERN CENTRAL ASIA

Perle Guarino-Vignon*[1], Nina Marchi[1,2], Julio Bendezu-Sarmiento[1,3], Evelyne Heyer[1], Céline Bon[1]

[1] *UMR7206 Eco-anthropologie, CNRS - MNHN – Université de Paris - Sorbonne Paris Cité, Paris, France*

[2] *CMPG, Institute of Ecology and Evolution, University of Berne, Switzerland*

[3] *Délégation archéologique française en Afghanistan*

**Corresponding author*

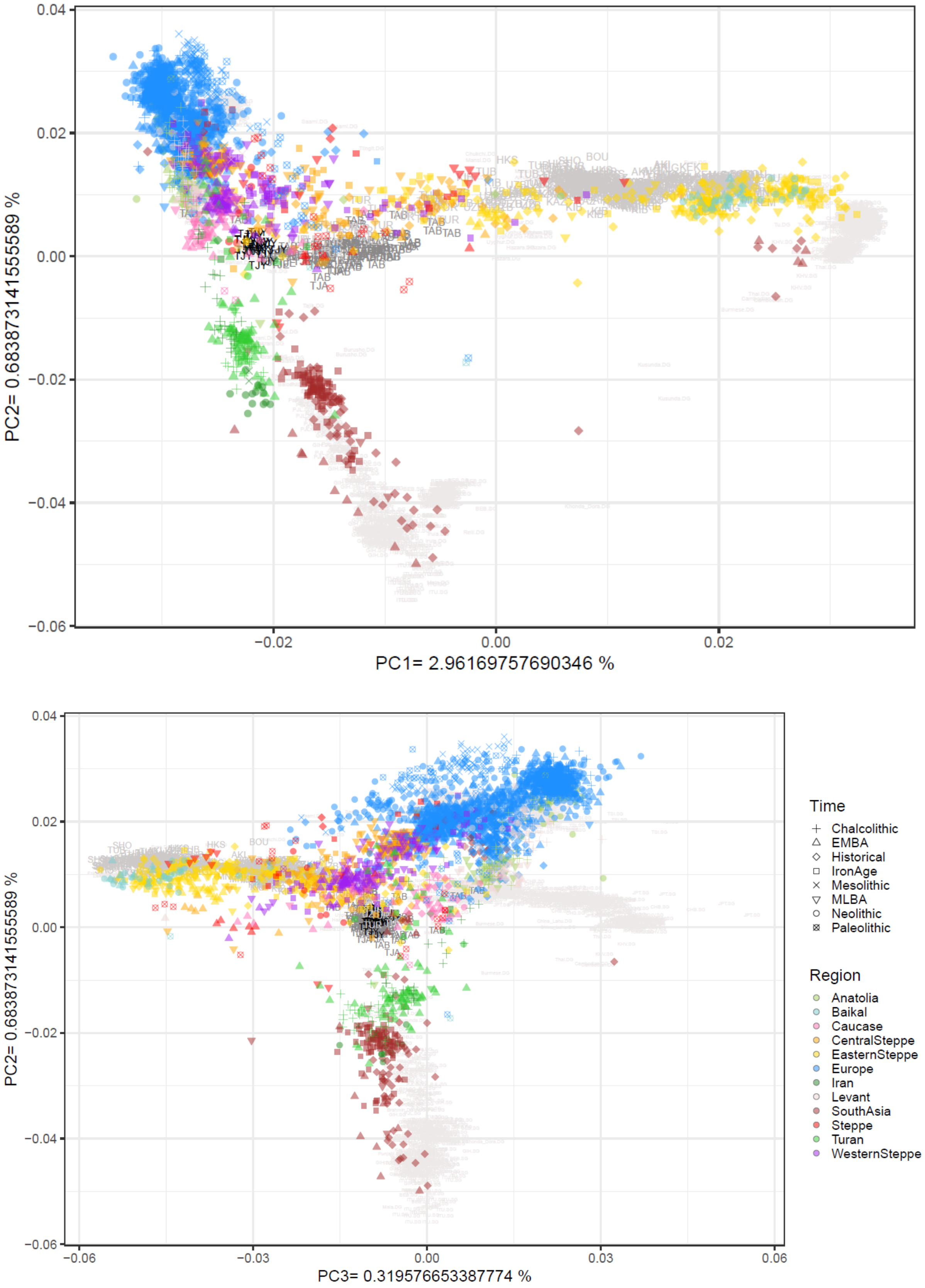

Figure S1. Full PCA computed on 1915 modern genomes, with projection of 3102 ancient genomes with *smartpca*. (Figures done with ggplot2 v. 3.3.3 R package https://cran.r-project.org/web/packages/ggplot2/index.html)

LASER

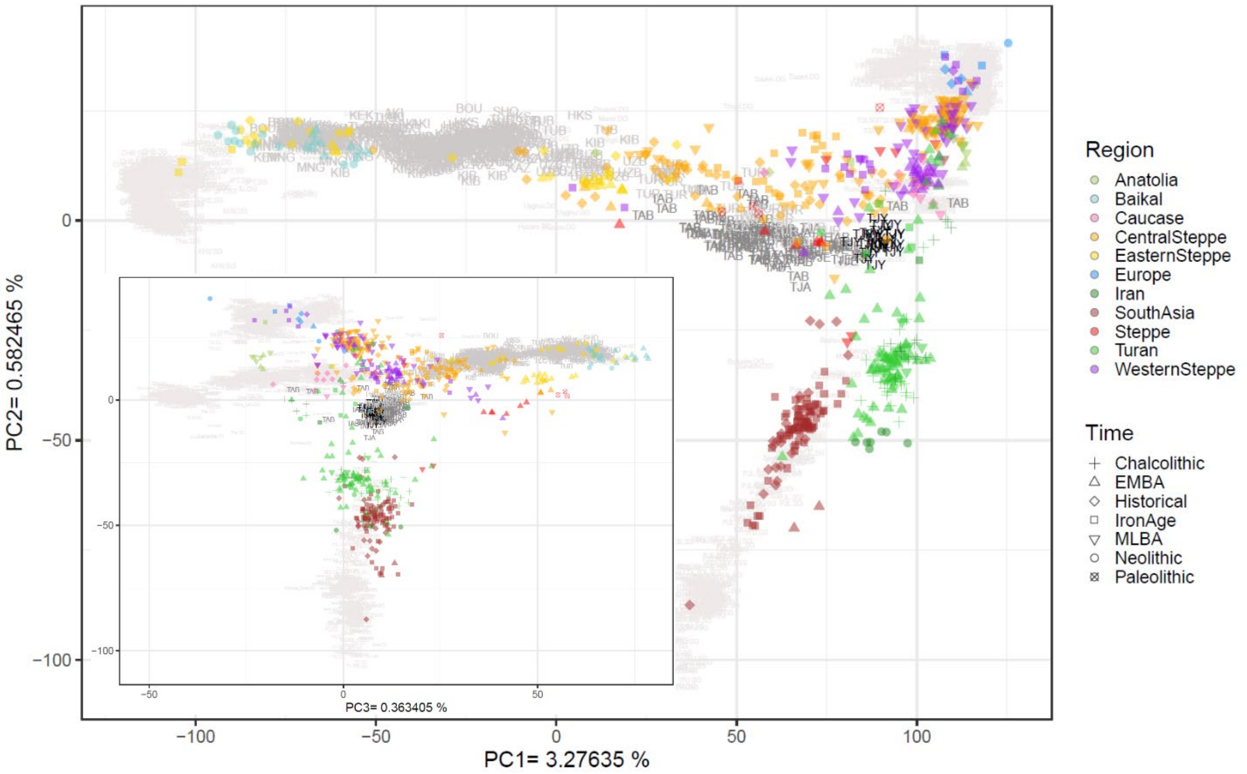

smartpca

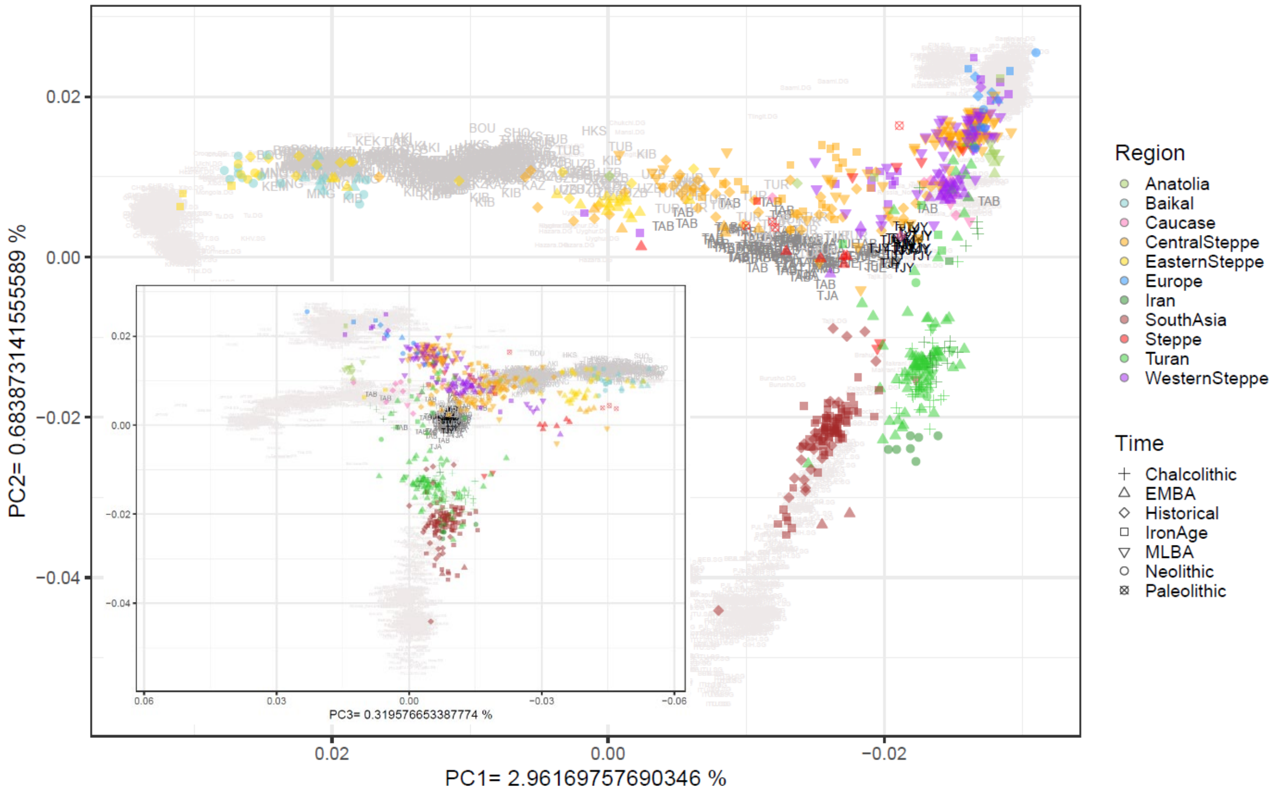

Figure S2.A – Top: PCA computed on 1915 modern genomes, with projection of 635 ancient genomes with *LASER*. **Bottom:** PCA computed on 1915 modern genomes, with projection of the same 635 ancient genomes with *smartpca*. (Figures done with ggplot2 v. 3.3.3 R package https://cran.r-project.org/web/packages/ggplot2/index.html)

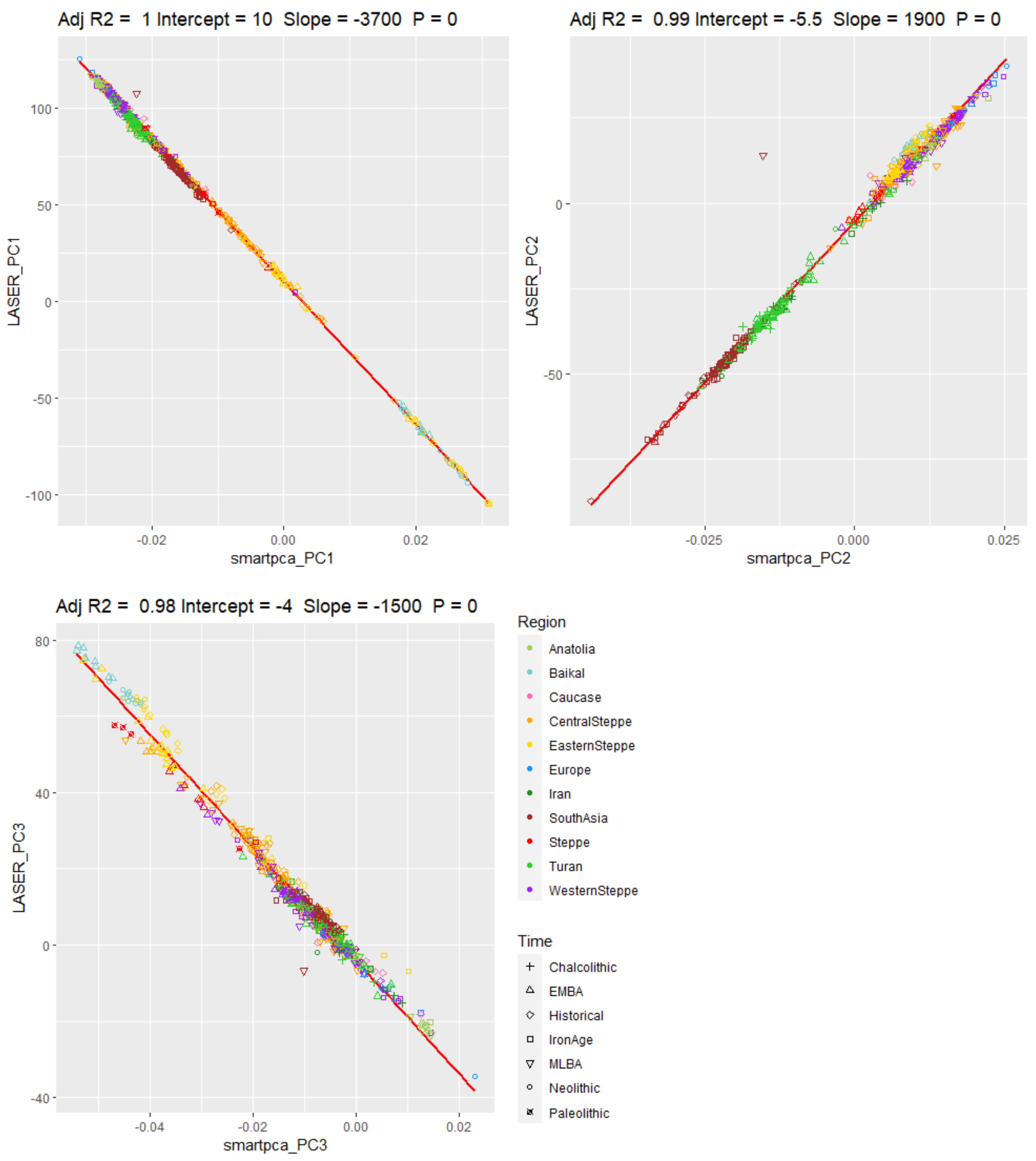

Figure S2.B – Correlation between the position of the ancient individuals on the PCs calculated by LASER and *smartpca*.

(Figures done with ggplot2 v. 3.3.3 R package https://cran.r-project.org/web/packages/ggplot2/index.html)

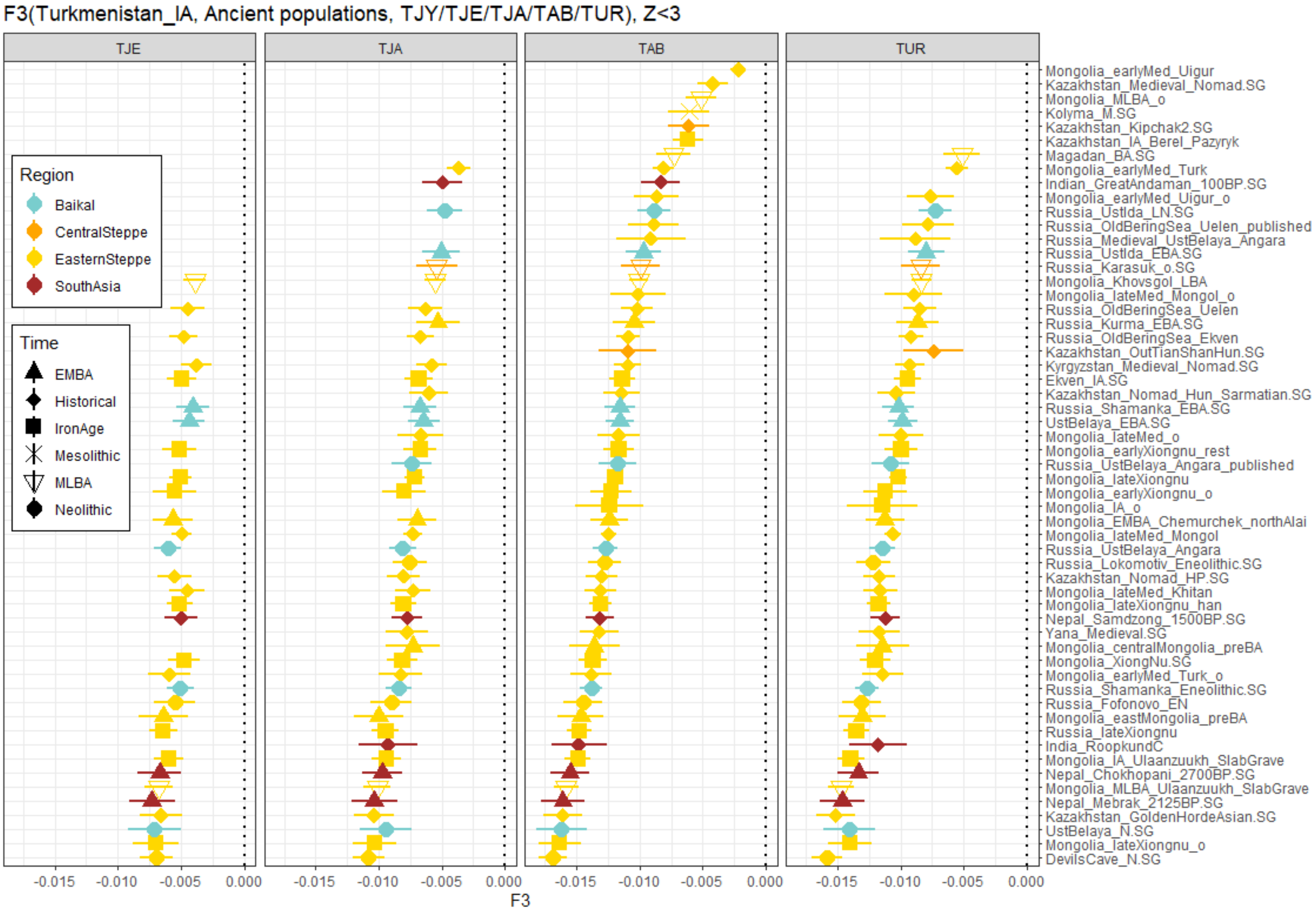

Figure S2. – All the significatively negative admixture f3-statistics in the form f3(*Turkmenistan_IA*, *Ancient population*, TJY/TJE/TJA/TAB/TUR) calculated on the first dataset. We did not obtain significative f3 for TJY. (Figure done with ggplot2 v. 3.3.3 R package https://cran.r-project.org/web/packages/ggplot2/index.html)

Figure S3 – Full ADMIXTURE analysis for all ancient individuals

(Figure done with ggplot2 v. 3.3.3 R package https://cran.r-project.org/web/packages/ggplot2/index.html)

Figure S4 – Full ADMIXTURE analysis for all present-day individuals (Figure done with ggplot2 v. 3.3.3 R package https://cran.r-project.org/web/packages/ggplot2/index.html)

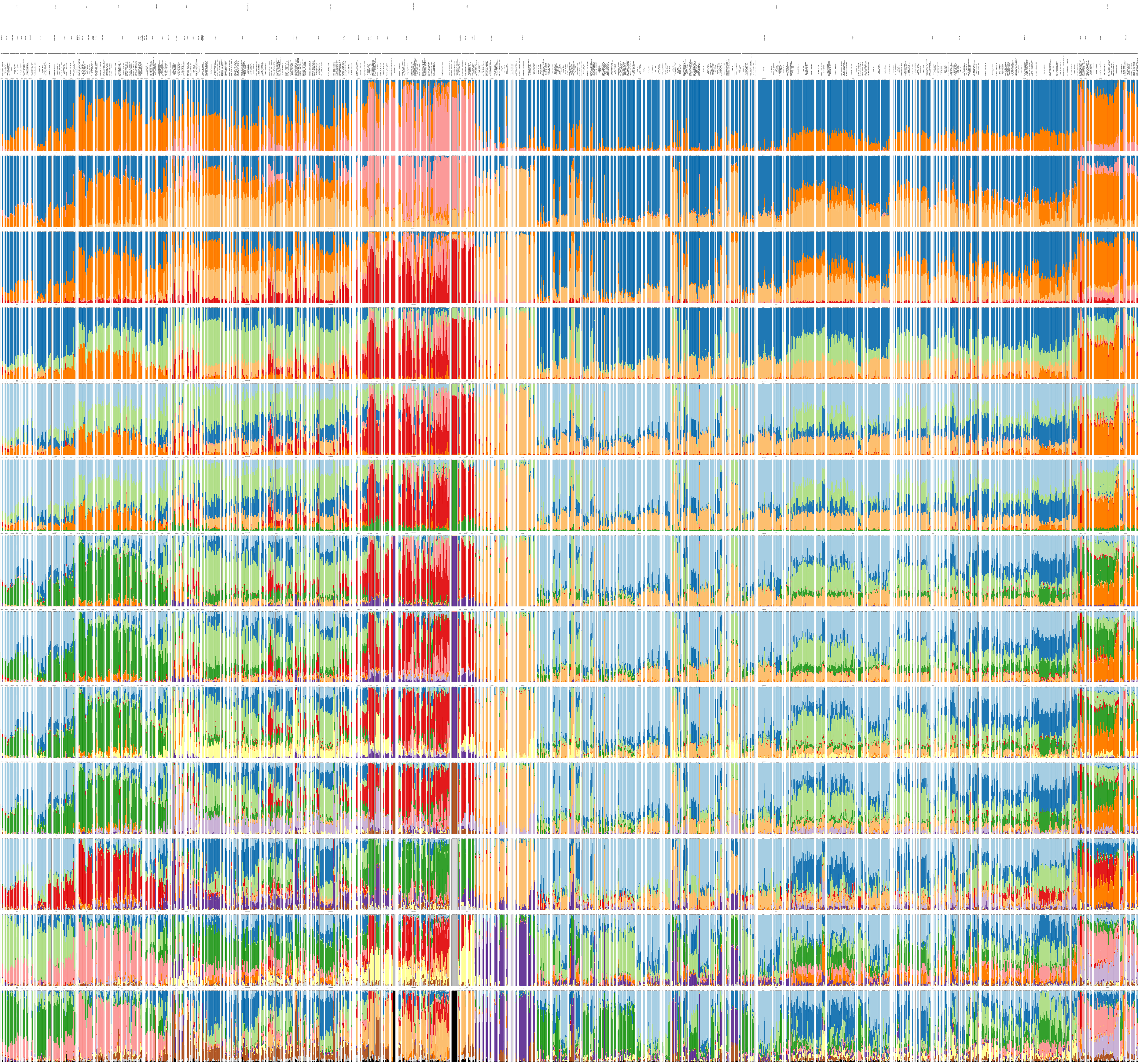

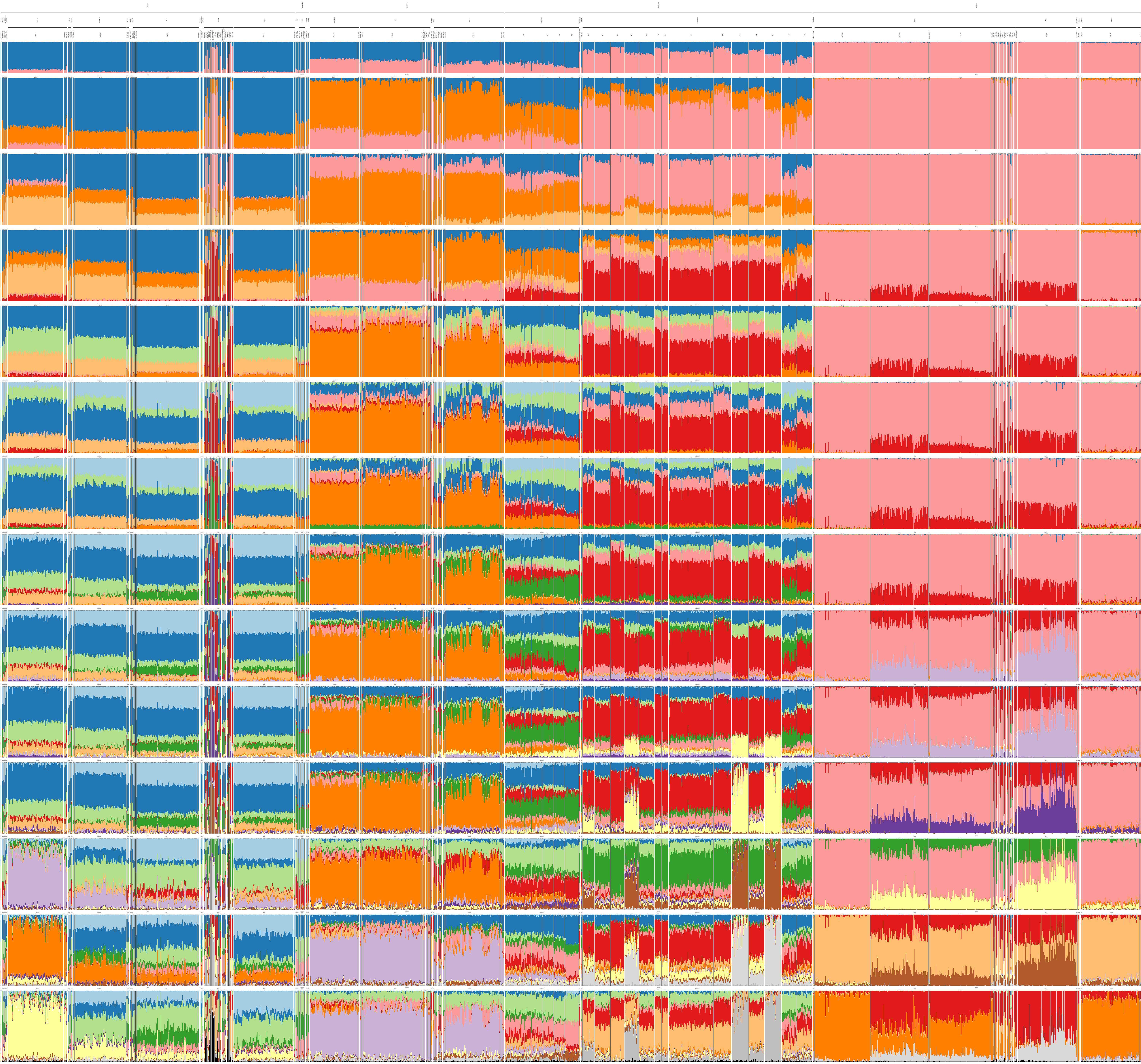
Europe
MiddleEast
SouthAsia

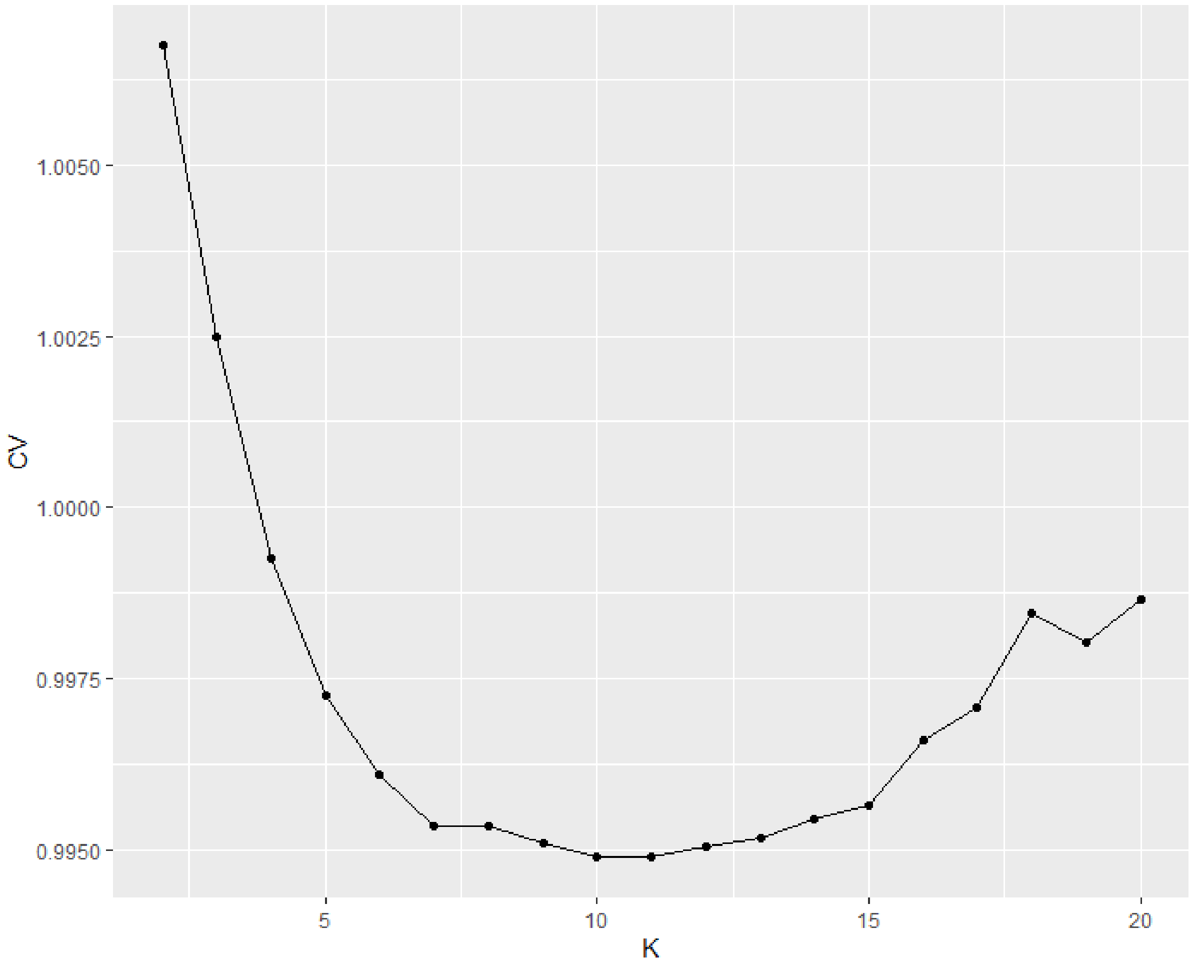

Figure S6 – Cross validation plot for ADMIXTURE analysis (Figures done with ggplot2 v. 3.3.3 R package https://cran.r-project.org/web/packages/ggplot2/index.html)

Table S1 – Contextual information for published ancient genome-wide data

Table S2 – Contextual information for published modern genomes from all Eurasia

Table S3 – Contextual information for modern genome-wide data from Central Asia (300k SNPs array)

Table S4 – Contextual information for modern genome from Central Asia (Shotgun)

Table S5 – All D-stat of the form D(Mbuti, Ancient pop; Iron Age Turan, TAB/TJA/TJE/TJY/TUR)

Table S6 – All f3-admixture of the form f3(Ancient pop, Iron Age Turan; TAB/TJA/TJE/TJY/TUR)

Table S7 - Significantly negative f3-admixture of the form f3(Ancient Pop1, Ancient Pop2; TJY/TJE/TJA/TAB)

Table S8 – All f3-outgroup of the form f3(Ancient pop, Central Asia pop; Outgroup)
